# P03-010 - IL10 SNPs associated with BD in Western Algeria

**DOI:** 10.1186/1546-0096-11-S1-A205

**Published:** 2013-11-08

**Authors:** O Khaib Dit Naib, M Aribi, A Idder, A Chiali, H Sairi, I Touitou, G Lefranc, M Barat-Houari

**Affiliations:** 1 Immunology, Tlemcen University, Tlemcen, Alger; 2 Ophtalmology, Hamou Boutlelis Clinic, Alger; 3 Dermatology, Chu, Oran, Algeria; 4 INM U844, Inserm, France; 5 Montpellier University, France; 6 Genetic, Chru de Montpellier, France; 7 Immunogenetic, CNRS UPR1142 IGH, France; 8 Genetic, Chru, Montpellier, France

## Introduction

Behcet's disease (BD) is a multisystem inflammatory disease, characterized by recurrent, oral and genital ulceration, skin lesions and uveitis. Several publications in the last decades showed the complex role of genetic factors; recent studies have revealed that SNPs of the *IL10* gene promoter are associated with BD in various populations.

## Objectives

We aimed to test the hypothesis that two SNPs of the *IL10* gene promoter (c.-819C>T, rs1800871 and c.-592C>A, rs1800872) may act as predisposing factors for BD in Algerian patients.

## Methods

Fifty one BD patients and 96 unrelated controls from Western Algeria were genotyped for the two SNPs by direct sequencing. Allele and genotype distributions were compared between cases and controls, using Chi2 or Fisher's exact tests.

## Results

The minor alleles c.-819T and c.-592A, were significantly more frequent (i) in BD patients than in controls (44% versus 27%, *p*= 0.003, OR= 2.18; 95% CI 1.33, 3.90) and (ii) in patients with genital ulcers or skin lesions than those without (OR=2.28, *p*= 0.002, 95% CI 1.10, 1.60 and OR = 2.18, *p*= 0.0035, 95% CI 1.27, 3.72, respectively).

## Conclusion

Our results showed that two investigated SNPs play a role in BD and in most of its related phenotypes in the population of Western Algeria. These observations are consistent with those reported for other ethnic groups, but need to be confirmed in a larger sample.

## Competing interests

None Declared.

